# Detection of alleles associated with resistance to chemical insecticide in the malaria vector *Anopheles arabiensis* in Santiago, Cabo Verde

**DOI:** 10.1186/s12936-019-2757-3

**Published:** 2019-04-05

**Authors:** Derciliano Lopes da Cruz, Marcelo Henrique Santos Paiva, Duschinka Ribeiro Duarte Guedes, Joana Alves, Lara Ferrero Gómez, Constância Flávia Junqueira Ayres

**Affiliations:** 1 Departamento de Entomologia, Instituto Aggeu Magalhães/Fundação Oswaldo Cruz (FIOCRUZ-PE), Av. Professor Moraes Rego s/n, Cidade Universitária, Recife, PE 50670-420 Brazil; 2 Universidade Federal de Pernambuco, Centro Acadêmico do Agreste, Rodovia BR-104, km 59 - Nova Caruaru, Caruaru, PE 55002-970 Brazil; 3 Instituto Nacional de Saúde Pública/Ministério da Saúde, Largo do Desastre da Assistência, CP-719, Praia, Cabo Verde; 4 Universidade Jean Piaget (UniPiaget), Caixa Postal 775, Praia, Cabo Verde

**Keywords:** *Anopheles arabiensis*, Insecticide resistance, L1014S kdr allele, *GSTE2*

## Abstract

**Background:**

Mosquitoes of the *Anopheles gambiae* complex are the main malaria vectors worldwide. Due to the lack of a vaccine to prevent malaria, the principal way to reduce the impact of this disease relies on the use of chemical insecticides to control its vectors. However, the intensive use of such compounds has led to the emergence of insecticide resistance in several *Anopheles* populations in Africa. This study aimed to investigate the presence of resistance alleles in an *Anopheles arabiensis* population from the City of Praia, capital of the Archipelago Cabo Verde, one of the countries on the World Health Organization list of countries that are on a path to eliminate local transmission of malaria.

**Methods:**

Larvae from the *Anopheles* genus were collected using a one-pint dipper in three areas of City of Praia. Larvae were fed and maintained until the emergence of adult mosquitoes, and these were morphologically identified. In addition, molecular identification was performed using IGS markers and all *An. arabiensis* samples were subjected to PCR to screen for mutations associated to resistance in the *Ace*-*1*, *Na*_*v*_ and *GSTE2* genes.

**Results:**

From a total of 440 mosquitoes collected, 52.3% were morphologically identified as *An. gambiae* sensu lato (s.l.) and 46.7% as *Anopheles pretoriensis.* The molecular identification showed that 100% of the *An. gambiae* s.l. were *An. arabiensis.* The mutations G119S in the *Ace*-*1* gene and L119F in the *GSTE2* gene were screened but not found in any sample. However, sequencing analysis for *GSTE2* revealed the presence of 37 haplotypes, 16 polymorphic sites and a high genetic diversity (π = 2.67). The L1014S mutation in the *Na*_*v*_ (voltage-gated sodium channel gene) was detected at a frequency of 7.3%.

**Conclusion:**

This is the first study to investigate the circulation of insecticide resistance alleles in *An. arabiensis* from Cabo Verde. The circulation of the L1014S allele in the population of *An. arabiensis* in the city of Praia suggests that pyrethroid resistance may arise, be quickly selected, and may affect the process of malaria elimination in Cabo Verde. Molecular monitoring of resistance should continue in order to guide the development of strategies to be used in vector control in the study region.

## Background

Mosquitoes from the *Anopheles gambiae* complex are considered the main vectors of malaria, responsible for transmitting the protozoan parasite *Plasmodium* spp. to humans [1–3]. In Africa, most of the malaria transmission is sustained by members of the *An. gambiae* complex, in which *An. gambiae* sensu stricto (s.s.) is the most efficient and most studied vector in the African continent [4–6].

In West Africa, 374 million people are at risk of contracting malaria. In this region, malaria cases are mainly caused by *Plasmodium falciparum* (almost 100%) [7]. In the Cabo Verde archipelago, malaria arose in the fifteenth century, during the settlement of the islands, brought by immigrants from the western part of the Africa and Europe [8]. Currently, malaria in Cabo Verde is considered irregular, with a seasonal and sporadic transmission, low endemicity and quite variable from year to year, with no more than 100 cases per year [8, 9]. *Anopheles arabiensis*, a member of the *An. gambiae* complex, is the only known species in the Cabo Verde Archipelago, which is associated with the transmission of *Plasmodium*. This vector is well distributed in most of the islands of the country, except in Sal and Brava, and it has been responsible for major malaria outbreaks in the archipelago [10–13].

Due to the lack of malaria vaccines, control of this mosquito species has been an important strategy to prevent the disease. The use of long-lasting insecticidal nets (LLINs) and indoor residual spraying (IRS) is the main component of the strategies to prevent malaria. Currently, five classes of chemical insecticides are approved for using in IRS: pyrethroids (PYRs), organochlorides (OCs), organophosphates (OPs), carbamates (CMs) and pyrroles (e.g. chlorfenapyr) [14, 15].

In Cabo Verde, control strategies are performed in an integrated manner for both dengue and malaria vectors, *Aedes aegypti* and *An. arabiensis*, respectively, through the use of the OP larvicide temephos and IRS with the PYR deltamethrin [16]. In addition, adulterated gasoil and *Gambusia affinis* fish have also been used as control tools against mosquito larvae [17]. It is important to highlight that *Aedes aegypti* populations from Cabo Verde already displayed resistance to temephos and deltamethrin [18, 19]. In this context, it is necessary to investigate insecticide resistance in anopheles populations from Cabo Verde. Resistance to OP and PYR compounds have been associated with two major mechanisms: target-site insensitivity and metabolic detoxification [20, 21]. In general, for OP insecticides, the target-site insensitivity has been related to alterations in the acetylcholinesterase gene; while for PYR, polymorphisms in the para-gated sodium channel gene (*Na*_*v*_) have been implicated, which have been named *kdr* mutations (knockdown resistance) [22]. The metabolic resistance is based on the increase of the capacity of metabolizing chemical compounds, and it is generally associated with mutations in genes that codify members of three large families of detoxification enzymes: the cytochrome P450-dependent monooxygenases (MFOs or CYP450s), esterases, and glutathione *S*-transferases (GSTs) [23]. Mutations associated with insecticide resistance, such as the L119F in the *GSTE2* gene, G119S in the de *Ace*-*1* gene and the L1014F/L1014S in the *Na*_*v*_ gene, have been reported in several populations of *An. gambiae* sensu lato (s.l.) in Africa [24–32].

Cabo Verde is listed as one of the 21 countries in the world that aims to eliminate malaria by 2020 [13, 33], however, this action may be threatened due to the emergence of a high number of recent malaria cases reported in the country. For this reason, it is urgent to investigate the presence of alleles associated with resistance to chemical insecticides in natural populations of *An. arabiensis* circulating in the City of Praia, Santiago Island, Cabo Verde.

## Methods

### Study area

**Fig. 1:**
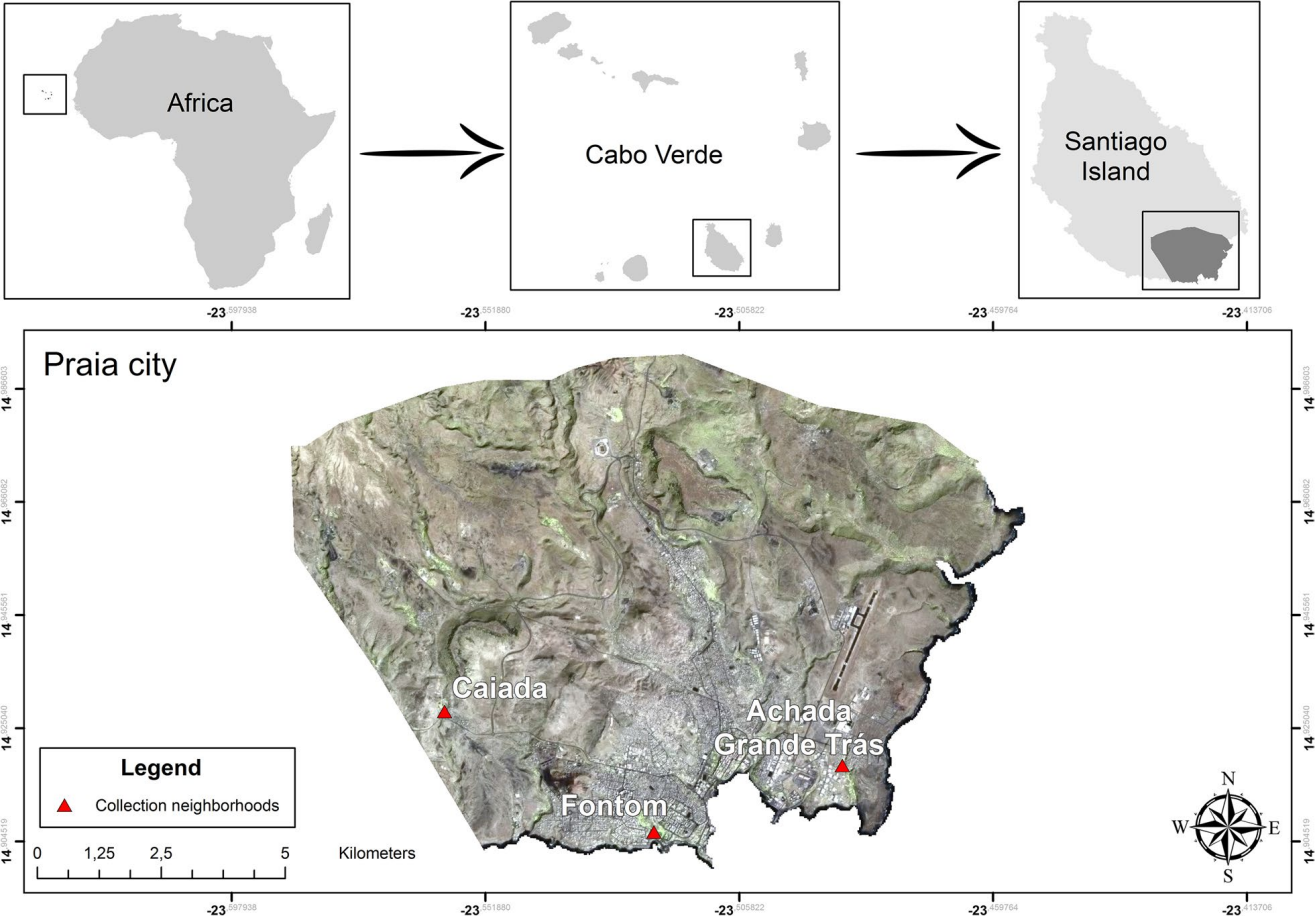
Geographic location of the Cabo Verde archipelago and the three sites where mosquitoes were collected (Source: edited in ArcGis, 2017)

This study was carried out in three neighborhoods of the City of Praia, island of Santiago, in Cabo Verde: Caiada (W023°33′33.40″, N14°55′41.24″), Achada Grande Trás (W023°29′13.53″, N14°55′5.94″) and Fontom (W023°31′16.64″, N14°54′22.61″) (Fig. 1).

Cabo Verde is an archipelago of volcanic origin situated in the Atlantic Ocean about 450 km off the West Coast of Africa, west of Dakar (Senegal), which occupies an area of 4033 km^2^. The resident population is around half a million inhabitants, with the majority located on the largest island of the archipelago, Santiago. The city of Praia, is the capital of the country and has the highest population density [34]. Caiada, located to the west of the city, is a semi-urban area with a large agricultural area. The population density is lower, compared to the localities of Fontom and Achada Grande Trás. Fontom, on the other hand, is located to the southeast, next to the seacoast, approximately seven kilometres from Caiada. It is an urban area with a higher population density compared to the other two locations. Achada Grande Trás, also considered an urban area, lies to the east of the city of Praia, approximately 4 km from the locality of Fontom.

### Mosquito collection

During September 2015, larvae of the *Anopheles* genus were collected from natural and artificial breeding sites such as puddles and open water storage tanks using one-pint dippers. Samples were taken to the Laboratory of Entomology at the Jean Piaget University of Cabo Verde, separated by location and kept at rearing trays with water and fish food (*Nutra Fish*^*®*^), under standard conditions (25–28 °C, 65–75% relative humidity and 12 h cycles of light/dark). Pupae were transferred to cages for adult emergence, and mosquitoes were fed with 10% sucrose solution and sterilized water for up to 3 days. Individuals were transferred to 1.5 ml microtubes and stored at − 20 °C for 30–40 min for immobilization and subsequent morphological identification.

### Morphological identification and DNA extraction

Adult mosquitoes from the *Anopheles* genus were identified using a morphological identification key [12]. A total of 501 mosquitoes were identified morphologically, however, only 440 mosquitoes were considered in this study. After identification, mosquitoes were kept in 70% ethanol until DNA extraction. All DNA was extracted from individual mosquitoes according to the protocol of Ayres et al. [35], quantified using Nanodrop 2000 (ThermoScientific), and stored at − 20 °C.

### Molecular identification of species from the *Anopheles gambiae* complex

Species identification of *An. gambiae* complex was performed using previously reported specific primers that amplify the IGS (intergenic spacers) region [36, 37]. Distinction between species of the *An. gambiae* complex was conducted according to the length of the amplicons as follows: 153 bp for *Anopheles quadrianulatus*, 315 bp for *An. arabiensis*, 390 bp for *An. gambiae* s.s., 464 bp for *Anopheles melas* and 466 bp for *Anopheles merus* [37]. *Anopheles gambiae* s.s. DNA samples, kindly provided by Dr. Maria Helena Silva Filha (Entomology Department at FIOCRUZ-PE), were used as positive controls. A batch of samples (nine) was submitted for DNA sequencing to confirm its identity using an ABI 3500 × L Applied Biosystems in the Nucleus of Technology Platform Fiocruz. After sequencing, the identity of the samples was confirmed using nucleotide BLAST (BLASTn) from the National Center for Biotechnology Information (NCBI) database (https://blast.ncbi.nlm.nih.gov/Blast.cgi).

### Screening of alleles associated to chemical insecticides resistance

After the species molecular identification, samples were used for the screening of resistance alleles linked to the use of chemical insecticides through PCR assay. The genes investigated were: acetylcholinesterase (*Ace*-*1*) (AGAP001356), *GSTE2* (AARA008732), a member of the glutathione *S*-transferases epsilon class (GSTs) and voltage-gated sodium channel gene (*Na*_*v*_) (KR867649.1) family.

### Detection of *Ace*-*1* mutations

A PCR–RFLP was used to screen for the G119S mutation in the *Ace*-*1* gene, from previously published protocol [38]. A 541-bp DNA fragment was amplified using the PCR GoTaq^®^ Flexi DNA polymerase kit (Promega, USA) in a total volume of 25 μl containing 10 µM of each primer Ex3AGdir 5′GATCGTGGACACCGTGTTCG3′ and Ex3AGrev 5′AG-GATGGCCCGCTGGAACAG3′. For digestion reaction, 7.5 µl of PCR product were used with 5U of *Alu*I enzyme (New England Biolab) in a final volume of 25 µl and incubated at 37 °C for three hours. Digestion products were analysed by electrophoresis in a 2% agarose gel. Susceptible homozygous (SS) individuals must display, after electrophoresis run, 403 bp and 153 bp long fragments, and two fragments of 253 bp and 150 bp for resistant homozygous (RR). The heterozygous (RS) show a combination of susceptible and homozygous resistant bands [25].

### Detection of mutations in the *GSTE2* gene and genetic diversity

Specific primers (*forward* 5′-AGTTCGCTGCGAAAATGTCC-3′ and *reverse* 5′-CCAAATGCTTCCAAATTTAACTC-3′) were used to detect the L119F mutation [39]. The amplified fragment of 895 bp comprises part of exon I up to part of exon III and the two complete introns. PCRs were performed using PCR GoTaq^®^ Flexi DNA polymerase (Promega, USA) kit according to the manufacturer’s recommendations, and thermocycling conditions were as follows: denaturation at 94 °C for two minutes, 35 cycles of denaturation at 94 °C for one minute, annealing at 52 °C for one minute, and extension at 72 °C for 1 min; and a final extension at 72 °C for 5 min. PCR products were visualized in 1% agarose gels under UV light and sequenced for both directions in an ABI 3500 × L Applied Biosystems in the Nucleus of Technology Platform Fiocruz.

Sequences obtained for the *GSTE2* gene fragment were screened for other mutations, besides the L119F. The polymorphic sites were identified using BioEdit, where multiple alignments were performed using ClustalW [40]. All sequences were aligned versus the full *GSTE2* genomic sequence obtained from VectorBase, (http://www.vectorbase.org/) and synonymous and non-synonymous polymorphisms identified. DnaSP 5.10.01 was used to obtain the genetic diversity indices [number of haplotypes, haplotype diversity and nucleotide diversity (π)] and neutrality test (D and D* selection estimates) [41].

### Detection of mutations in the *Na*_*v*_ gene

For the screening of L1014F/L1014S mutation, a 458 bp fragment was amplified using forward (5′-TTTACAATGCCAACGCAATC-3′) and reverse (5′ GATCTTGGTCCATGTTAATTTGC-3′). These primers were designed using the Primer3 software (sequence access number in GenBank: KR867649.1). PCR was performed using the GoTaq^®^ Flexi DNA polymerase PCR kit (Promega, USA), according to the manufacturer’s instructions. Amplification was performed under the following conditions: initial denaturation at 95 °C for 2 min and 35 cycles of 94 °C for 30 s, 55 °C for 30 s, 72 °C for 30 s and 72 °C for 5 min. PCR products were analysed in 1.5% agarose gel electrophoresis and visualized in U.V. trans-illuminator. All PCR products were sequenced in both directions in an ABI 3500 × L Applied Biosystems.

### Sequence analysis

PCR products of the genes that were analysed (*GSTE2*, *Na*_*v*_, *Ace*-*1*) were sent for sequencing at the Nucleus of Technology Platforms (NPT) of IAM. However, for *Ace*-1 gene only ten random samples were selected for sequencing. All the obtained sequences were analysed in the CodonCode Aligner Program (version 4.7) for sequence quality assessment, editing and assembling of contigs (assembly criteria: sequences with quality ≥ 20 were used to generate the consensus sequences based on the PHRED program). Sequence alignment and mutation identification was performed in the BioEdit program (version 7.2.6).

## Results

### Morphological and molecular identification of species from the *Anopheles gambiae* complex

**Fig. 2:**
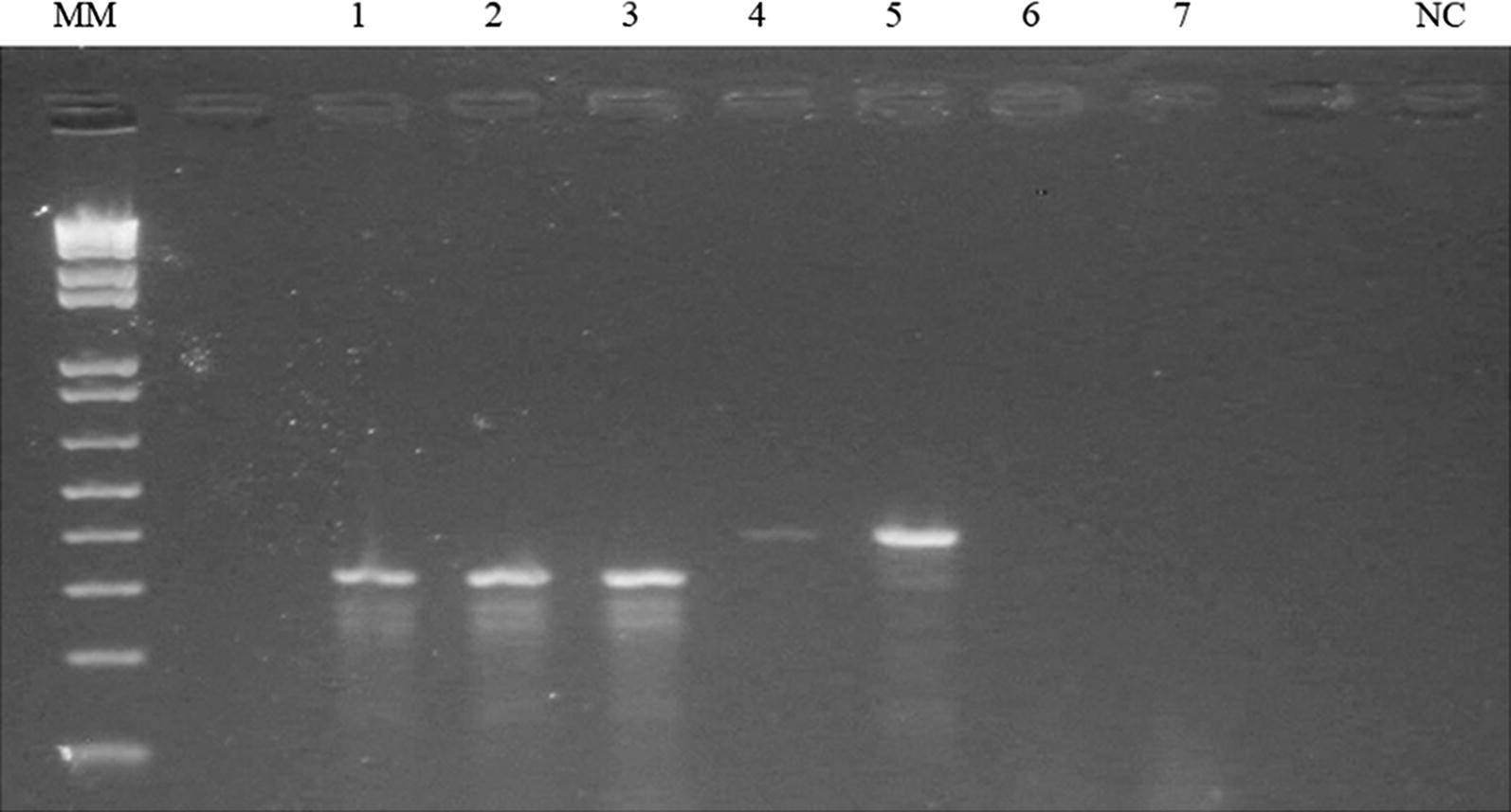
Amplification of the IGS region of mosquitoes from the *Anopheles gambiae* complex of the City of Praia. MM—molecular marker 1 Kb plus DNA *ladder*; NC—negative control; 1 to 3—*Anopheles arabiensis* (315 bp); 4 and 5—*Anopheles gambiae* s.s. (390 bp) used to show difference in size of bands among species of *Anopheles*; 6 and 7—DNA samples of *Anopheles pretoriensis* used as negative control

Out of 440 mosquitoes, 52.3% (230) were morphologically identified as *An. gambiae* s.l. and 46.7% (210) as *An. pretoriensis*. The *An. gambiae* s.l. samples were collected predominantly in the locality of Fontom (41.8%) followed by Caiada (8.0%) and Achada Grande Trás (2.5%). The molecular analysis identified 100% of the 230 individuals from the *An. gambiae* complex as *An. arabiensis* (Fig. 2). Nine random PCR products were submitted to sequencing and identified as *An. arabiensis*.

### Screening of alleles associated with chemical insecticides resistance

#### Screening for *Ace*-*1R* mutation

The G119S mutation was not detected in any of the 230 *An. arabiensis* samples investigated. RFLP-PCR using the restriction enzyme *Alu*I revealed that all individuals are homozygous susceptible (SS). Sequencing analysis of randomly chosen samples also confirmed the absence of the G119S mutation in *An. arabiensis* population from Praia.

#### Detection of mutations in the *GSTE2* gene and genetic diversity

**Fig. 3:**
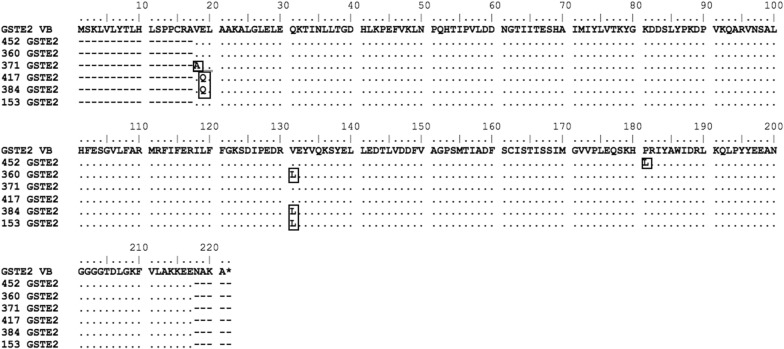
Amino acid alignment of *GSTE2* coding sequence of *Anopheles arabiensis*. *GSTE2* VB is the coding sequence from the *Anopheles arabiensis* genome sequence (Gene identifier AARA008732 on http://www.vectorbase.com). Residues differing from the VectorBase sequence are highlighted

All *An. arabiensis* samples were screened for the identification of L119F mutation and sequencing was successful in 215 individuals. Although the L119F mutation was not found, the analysis of the *GSTE2* ORF fragment revealed the presence of other mutations: four non synonymous substitutions (V18A, E19Q, V131L, P181L) and two synonymous mutations. Non-synonymous mutations were detected in 33.5% of individuals, and the V131L was the most common and present in all localities. Individuals from Fontom exhibited the highest number of non-synonymous mutations (Table 1 and Fig. 3).

**Table 1:** Non-synonymous substitutions of nucleotides in the *GSTE2* ORF of *Anopheles arabiensis* specimens, distributed by locations in the City of Praia

ORF position^a^	Non-synonymous		Localities/number of individuals		
	Substitutions		Fontom	Caiada	AGT
18	T (Val)	C (Ala)	5	0	0
19	G (Glu)	C (Gln)	8	0	0
131	G (Val)	C (Leu)	49	3	4
181	C (Pro)	T (Leu)	3	0	0
Total	–	–	65	3	4

^a^AARA008732 vector base; *AGT* Achada Grande Trás

Sequence analysis of the 763 bp *GSTE2* gene (600 bp for 3 exons and 163 pb for 2 introns), revealed the presence of 37 haplotypes (GenBank accession numbers: from MG970292 to MG970322), 16 polymorphic sites (10 sites in the introns and six sites in the exons) and a nucleotide diversity of 2.67 (Table 2). The analysis of neutrality tests (Tajima’s D) was negative but not statistically significance (p > 0.10). Fu and Li’s test were positive and statistically significant (p-value < 0.05).

**Table 2:** Genetic parameters and tests of neutrality, for the coding and non-coding regions of *GSTE2* of *Anopheles arabiensis* mosquitoes of the City of Praia

Sequence size	Genetic parameters and neutrality indices						
	S	*h*	hd	π	D	D*	θ
Complete sequence (763 bp)	16	37	0.757	2.67	− 0.605	1.644*	3.16
Coding sequence (600 bp)	6	11	0.499	1.01	− 0.830	1.08	1.51
Non-coding sequence (163 bp)	10	21	0.722	8.81	− 0.292	1.3	9.24

*S* number of polymorphic sites, *h* number of haplotypes, *hd* haplotype diversity, *π* nucleotide diversity multiplied by 10^3^, *D* Tajima’s test statistic, D* Fu and Li’s test statistic

* Statistically significant. p < 0.05; θ, Watterson’s estimator (per site) multiplied by 10^3^

#### Detection of *kdr* mutations in *Na*_*v*_ gene

**Fig. 4:**
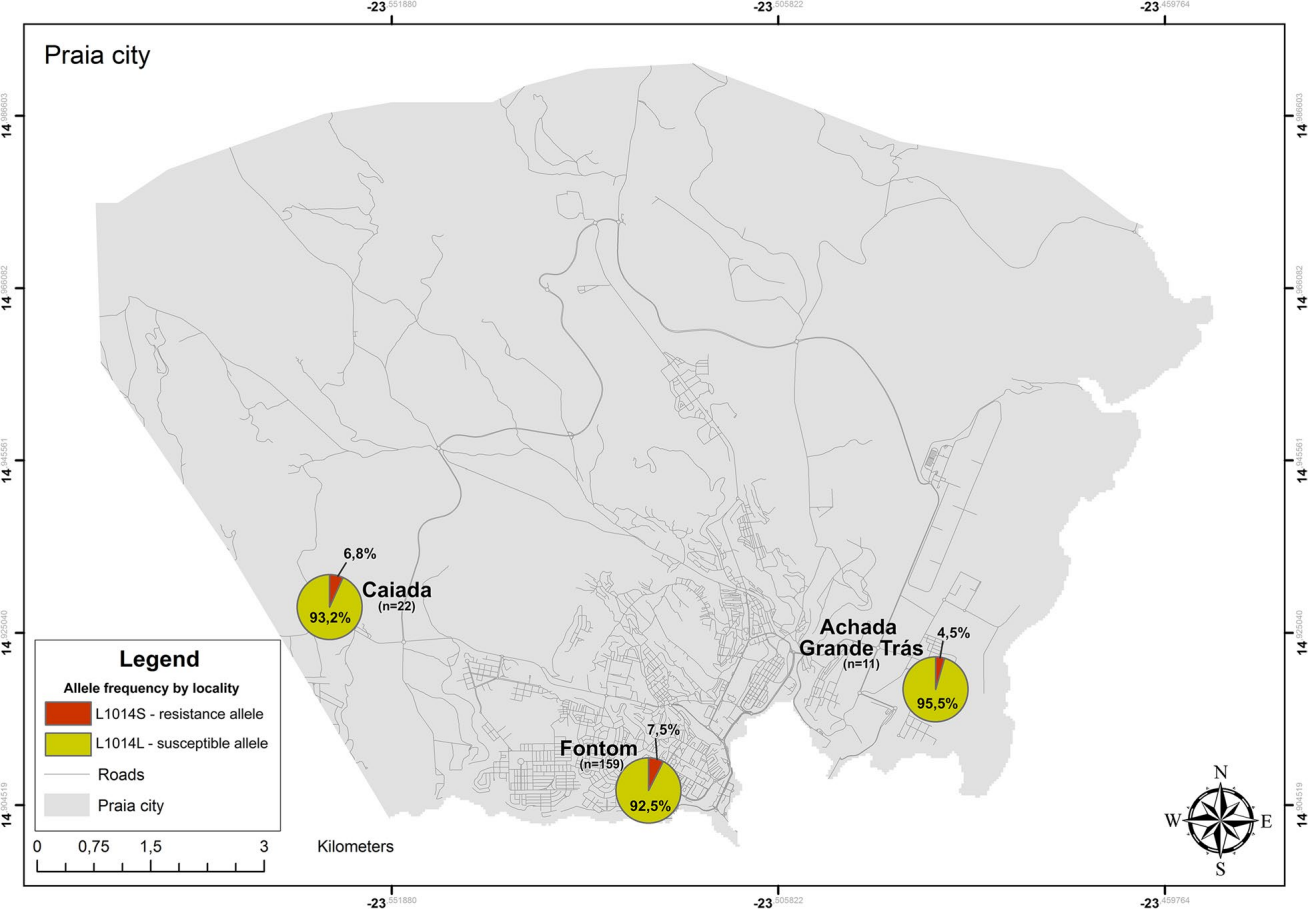
Allelic frequency distribution of L1014S of the *Na*_*v*_ gene at the collection sites, in the city of Praia, Cabo Verde. n is the total number of individuals used for detection of L1014S/L1014L mutations in the *Na*_*v*_ gene

Out of 201 individuals of the species *An. arabiensis* sequenced for the *Na*_*v*_ gene, 192 were genotyped to search for *kdr*-east (L1014S) and *kdr*-west (L1014F) mutations. Out of the 192 specimens analysed, 26 were heterozygous (RS) and one was homozygous (RR) for the L1014S mutation. The remaining (165) were homozygous for the susceptible genotype (SS). Sequences were submitted to the GenBank as follows: RR (MG970325), RS (MG970324), and SS (MG970323). The allelic frequency of L1014S was 7.3% (Table 3) and was more frequent in individuals collected in the Fontom neighbourhood (Fig. 4). The L1014F mutation was not detected in this population of analysed *An. arabiensis*, showing that all individuals analysed for this mutation were susceptible homozygous (SS).

**Table 3:** Genotype and allelic frequency (1014S) of the *Anopheles arabiensis* population at different collection sites in city of Praia

Localities	N	Genotypes			Allelic frequency	
		RR	RS	SS	R	S
Fontom	159	1	22	136	0.075	0.925
Caiada	22	0	3	19	0.068	0.932
AGT	11	0	1	10	0.045	0.955
Total	192	1	26	165	0.073	0.927

*N* number of mosquitoes, *R* resistant 1014S allele, *S* susceptible wild type allele, *RR* resistant homozygous individuals, *RS* heterozygous individuals, *SS* susceptible homozygous individuals

## Discussion

In the global context, Cabo Verde is among the 21 countries targeting the elimination of malaria by 2020 [33]. Although different strategies are employed in vector control programs, the use of chemical insecticides is still a key component in the context of malaria elimination.

The present study conducted for the first time a screening of resistance alleles in the malaria vector *An. arabiensis* from Cabo Verde. It is important to point out that this type of study had already been conducted in Cabo Verde with populations of *Aedes aegypti* resistant to organophosphate and pyrethroids [18]. Once the vector control strategies are conducted in an integrated manner, targeting both *Anopheles* and *Aedes* species, the same effect could be expected in the two species, once both have been exposed for the same period.

As confirmed by molecular taxonomic identification, *An. arabiensis* is the only species of the *An. gambiae* complex identified so far, as described by Cambournac et al. [11] and Ribeiro et al. [12]. However, it is necessary to perform an entomological survey with a greater spatial coverage in order to obtain a more accurate distribution panorama of *An. arabiensis* in Cabo Verde.

The absence of the two of the main mutations associated with chemical insecticides resistance described in *Anopheles* spp.: *Ace*-*1R* by insensitivity of target site and L119F-*GSTE2* by metabolic resistance, suggests a non-selection of these alleles that affects the functionality of acetylcholinesterase and *GSTE2*, respectively, but does not rule out the possibility of loss of susceptibility by alteration of other detoxification systems such as the monooxygenase family, esterases or other enzymes of the glutathione transferases family [42]. Therefore, biochemical tests in order to evaluate the activity of these detoxification enzymes could be performed in *An. arabiensis* populations from Cabo Verde. Concerning GSTs, association between high GST activity and resistance to organophosphates, organochlorines and pyrethroids in many insect species has been reported [43]. Yahouédo et al. [30] observed elevated levels of GST in *Anopheles* populations resistant to pyrethroid from Benin. Cisse et al. [44] reported high levels of GSTs conferring resistance to pyrethroid and DDT in *An. gambiae* s.l. in Mali.

In the present study, analysis of the *GSTE2* DNA sequences revealed an abundance of polymorphisms including non-synonymous mutations in the coding region of the gene. Future investigations, such as gene expression and molecular *docking*, should be carried out to verify if these mutations found in the coding region could play an important role in insecticide resistance [45]. Previous studies of molecular *docking* with AgGSTE2 and DDT have shown that the residues, Glu116, Phe120, Arg112 and Leu36 are considered important components of the AgGSTE2 site-active in the direct interaction with DDT. These residues act as a pocket that interact hydrophobically with DDT [46, 47]. The mutations observed in the present study do not correspond to any of these sites mentioned previously. On the other hand, two amino acid substitutions observed here (V18A and E19Q) are located in a highly conserved domain (between position 16 and 30 of the encoded protein) among all members of epsilon class GSTs in several species of *Anopheles* [48], which may indicate a selective process representing specific adaptations for this mosquito species. Recently, Mitchell et al. [49], through molecular modelling, described a variant *GSTE2*-114T that is significantly associated with DDT resistance in female mosquitoes of *An. gambiae* from West Africa. Pontes et al. [50] have demonstrated, in a molecular dynamics study, that AgGSTE2-F120L may be associated with DDT resistance in *An. gambiae.*

The high frequency of polymorphisms observed in *GSTE2* of the study population may be associated with a great variability of phenotypes. The high genetic diversity for this gene and significant positive value for the Fu and Li test (Table 2) may indicate that the *GSTE2* gene of the population studied may be under selection. On the other hand, the Tajima test was negative, not significant. Mirabello and Conn [51] also found negative Tajima D, but significant, in populations of *Anopheles darlingi* in South America and described this result as a recent expansion in population size after a bottleneck effect. The present data is not consistent with recent expansion; however, our sample size is probably low for analysing the demographic evolution of the *Anopheles* population. The selective process could be due to intensive use of insecticides in Praia, particularly in the localities of the study, among which is Fontom, considered as one of the hot spots for malaria transmission in the country and, therefore, receiving, in addition to routine integrated control carried out by the local programme, strengthening interventions made before and after the rainy season, using intra-domiciliar spraying. On the other hand, positive selection may also be associated with adaptation of larval stages to xenobiotics of the environment or other adverse conditions, such as temperature, dryness, among others as described in previous studies [52–54].

Comparing the population of *An. arabiensis* in Cabo Verde, based in *GSTE2* gene, with other *Anopheles* spp. vectors of the African continent, similar values for the genetic diversity indices were observed, for instance in Cameroon (hd = 0.89; π = 2.9), Mozambique (hd = 0.95; π = 3.6), and Malawi (hd = 0.78; π = 4.4). However, these indices are significantly higher when compared with those observed for populations of *Anopheles funestus* in Benin (hd: 0.088; π: 0.09) [39]. This wide range of genetic diversity for the same gene of detoxification in populations of *Anopheles* from the African continent can be explained by the geographical differences that exist among these populations.

Studies performed in different African countries have reported the presence of the G119S mutation in the *Ace*-*1* gene in *An. gambiae* s.l. populations, with allelic frequency variations ranging from 1 to 75% [26, 38, 55–58]. On the other hand, in some West and Central African countries such as Ghana, Burkina Faso and Cameroon, studies with mosquitoes of the *An. gambiae* complex revealed the absence of the G119S mutation in the populations studied [25, 29, 59]. The absence of the G119S mutation in the present study may indicate that the use of temephos in vector control programmes in Cabo Verde is not selecting alleles associated with target-site resistance to organophosphates in *An. gambiae* population.

Here, it was found, for the first time, the presence of the L1014S kdr allele in field populations of *An. arabiensis* in Cabo Verde, with a low allele frequency and in most cases in heterozygosis in the individuals evaluated (only one was homozygous). Although bioassays to assess the resistance phenotype were not performed, the presence of this allele suggests that resistance to pyrethroid may arise. The allele found can be quickly selected if no management is done regarding the use of pyrethroids through IRS. Therefore, bioassays are necessary to measure the phenotypic resistance of these mosquitoes to insecticides of the pyrethroids class (deltamethrin) used in vector control in Cabo Verde [60]. Recently, a brief report based on bioassays with samples of *An. gambiae* s.l. collected in Praia revealed tolerance to deltamethrin, corroborating our data [61]. Further investigation is also needed to determine the geographic distribution of the L1014S kdr allele and to evaluate its association with the use of chemical insecticides in regions where vector control is done with chemical insecticides [62]. In *An. arabiensis* populations, the L1014S allele has been found most frequently in East Africa, however, this mutation has been also reported in other West African countries such as Benin (allelic frequency ranging from 16 to 57%), Burkina Faso (range: 16% to 40%) and Kenya (range: 0.50% to 50%) [24, 28, 62–65]. These findings provide evidence of the spread of the L1014S allele in *An. gambiae* s.l. populations in West Africa.

Among the sites investigated in this study, Fontom was the district that presented the highest allelic frequency of L1014S. It is possible that there is an association between this high allelic frequency and the more frequent use of pyrethroids in this specific locality, unfortunately there is no public record of the use of this insecticide by locality. Other studies in African countries with anopheles populations have found a positive association between the high allelic frequency of L1014S and the frequent use of pyrethroids [66–68].

The L1014F allele has been reported in several African countries in mosquitoes of the *An. gambiae* complex, mainly in West Africa with allelic frequencies varying from 0.04 to 98% [69–72]. However, in this study this allele was not found in the population of *An. arabiensis* analysed. Likewise, in other African countries, such as Kenya and Burkina Faso, the L1014F allele was absent in mosquitoes of the *An. gambiae* complex [28, 64].

The results found in the present study are an alert for the competent authorities of Cabo Verde, since the L1014S allele was detected in the local *An. arabiensis* population in the archipelago for the first time. Especially at the current moment when the Cape Verdean Ministry of Health has invested in the use of impregnated mosquito nets to contain the malaria outbreak in the country.

There is an urgent need to create measures to avoid allele fixation in the analysed mosquito population. Therefore, the creation of a program to monitor the susceptibility of *An. arabiensis* becomes extremely important.

## Conclusion

This study aimed to investigate resistance alleles in *An. arabiensis* population, from City of Praia, Cabo Verde. The results obtained here show that no resistance-associated mutations in both *Ace*-*1* and *GSTE2* genes previously described for the *An. gambiae* complex were identified in the city of Praia. On the other hand, the *GSTE2* gene analysis revealed a high frequency of polymorphisms, which generate a high genetic diversity in the study population. Therefore, future studies should investigate the association of these new mutations with resistance to insecticides. Exon 20 analysis of the *Na*_*v*_ gene demonstrated the absence of the L1014F mutation and presence of L1014S allele at low frequency in these *An. arabiensis* populations from City of Praia.

These results serve as a basis and point out the need to conduct WHO bioassays to assess the susceptibility of *An. arabiensis* populations in Cabo Verde to the insecticides used locally, as well as the development of studies that elucidate what are the molecular mechanisms involved in the resistance. Monitoring the status of *An. arabiensis* population susceptibility to insecticides and the early detection of resistance allele’s dispersion can bring valuable subsidies for vector control in Cabo Verde, in the context of the 2020 pre-eradication malaria campaign.

## Abbreviations

*Ace*-*1*: acetylcholinesterase
DDT: dichlorodiphenyltrichloroethane
*GSTE2*: glutation *S*-transferase class epsilon 2
IRS: indoor residual spraying
*kdr*: knockdown resistance
LLINs: long-lasting insecticide nets
PCR: polymerase chain reaction
WHO: World Health Organization
AGT: Achada Grande Trás
*Na*_*v*_: voltage-gated sodium channel
